# Maculopapular rash with multiple drug hypersensitivity to cotrimoxazole, amikacin, piperacillin/tazobactam, and meropenem in a patient with hairy cell leukemia 

**DOI:** 10.5414/ALX02508E

**Published:** 2024-07-22

**Authors:** Katie Townsend, Claire Leck, Thippeswamy Billahalli, Elizabeth Barachina, Timothy J. Watts

**Affiliations:** 1 Department of Respiratory Medicine & Allergy, Homerton Healthcare NHS Foundation Trust,; 2 National Heart & Lung Institute, Imperial College London,; 3 Department of Immunology, Royal Free London Hospital NHS Foundation Trust, and; 4 University College London Institute of Immunity and Transplantation, Pears building, Rowland Street, London, United Kingdom

**Keywords:** multiple drug hypersensitivity, type IV hypersensitivity, delayed hypersensitivity, drug allergy

## Abstract

We describe a rare case of a 54-year-old female with hairy cell leukemia, who following treatment for neutropenic sepsis, developed an extensive severe maculopapular exanthema with perifollicular hemorrhage. Cladribine, cotrimoxazole, allopurinol, domperidone, amikacin, piperacillin/tazobactam, and meropenem had all been given in the 9 days prior to eruption onset. Three months later, drug patch testing/delayed intradermal testing was positive to cotrimoxazole, trimethoprim, amikacin, piperacillin/tazobactam, and meropenem, with additional evidence of penicillin cross-reactivity. Drug challenge tests were negative to allopurinol and domperidone. She was diagnosed with multiple drug hypersensitivity to cotrimoxazole, amikacin, piperacillin/tazobactam, and meropenem. Multiple drug hypersensitivity is a novel syndrome mainly seen with severe delayed type IV drug eruptions, involving long-lasting strong T-cell reactivity to two or more structurally unrelated drugs.

## Introduction 

Delayed type IV drug hypersensitivity reactions are characteristically T-cell mediated and include phenotypes ranging from milder maculopapular exanthemas (MPE) to more severe reactions (e.g., drug reaction with eosinophilia and systemic symptoms (DRESS)) [1]. Cross-reactivity between drugs causing delayed hypersensitivity is described in those with shared chemical properties (i.e., penicillins) [2]. In contrast, multiple drug hypersensitivity (MDH) is a more recently described novel syndrome involving long-lasting T-cell reactivity to two or more structurally unrelated drugs [1]. We describe a rare case of severe MPE with MDH to four different antibiotics. Informed consent was obtained from the patient for publication. 

## Case history 

**Figure 1.**
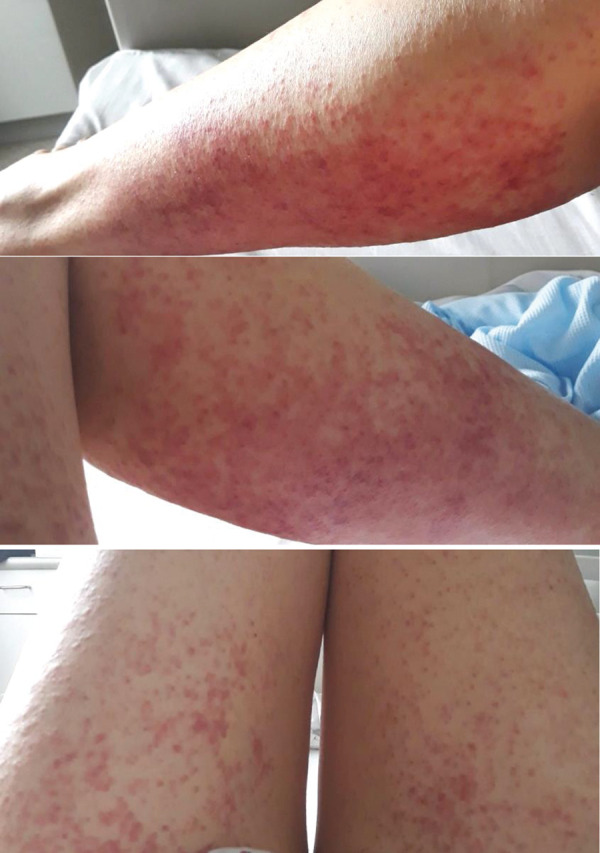
Generalized extensive maculopapular exanthema with perifollicular hemorrhage.

A 54-year-old female was referred to our drug allergy service for a suspected adverse drug reaction. She had been commenced on treatment for hairy cell leukemia with cladribine, allopurinol, domperidone, and cotrimoxazole; 7 days later she was hospitalized with fever. She was diagnosed with neutropenic sepsis (cladribine stopped) and started on amikacin, piperacillin/tazobactam, and meropenem. Approximately 48 hours later she developed facial erythema and edema; over the ensuing 72 hours this progressed cephalocaudally to a generalized severe extensive confluent maculopapular exanthema with perifollicular hemorrhage (Figure 1). There was no blistering, pustulosis, desquamation, or mucosal involvement. She had no prior history of dermatological or allergic conditions. Blood eosinophil count, liver and renal function all remained within normal limits. Skin biopsy showed superficial perivascular inflammation with subtle lichenoid interface-change and no eosinophil infiltrate, consistent with a morbilliform drug eruption. All drugs were discontinued ~ 48 hours post rash onset, and she gradually recovered over the ensuing 2 weeks with topical corticosteroids and emollients. 

## Results 

Three months later, drug patch testing was performed using IQ Ultra chambers (Chemo-technique Diagnostics, Vellinge, Sweden) with the commercialized forms of allopurinol (30% pet), domperidone (30% pet), penicillin V (30% pet), and trimethoprim (30% pet) – prepared using crushed tablets incorporated at 30% dilution in white petrolatum. Patch testing was also performed with the pure forms of cotrimoxazole (10% pet) and amoxicillin (10% pet), (via Chemo-technique Diagnostics) and using undiluted commercial drug solutions of amikacin (250 mg/mL), piperacillin/tazobactam (225 mg/mL), meropenem (50 mg/mL), and cladribine (2 mg/mL). Patch testing was conducted in accordance with European Society of Contact Dermatitis guidelines [3]; readings were at 48 hours and 96 hours as per International Contact Dermatitis Research Group Criteria [4]. 

**Figure 2.**
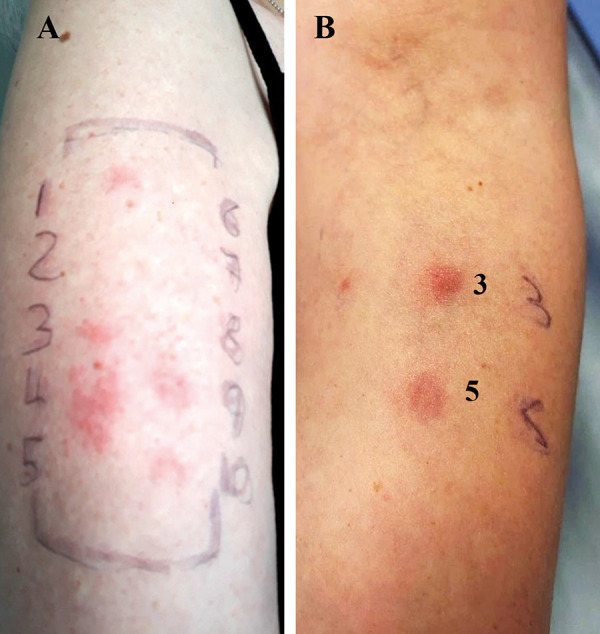
A: Positive patch test results at day 4, for: 1. cotrimoxazole (+), 3. amikacin (+), 4. piperacillin/tazobactam (++), 5. meropenem (+), 8. penicillin V (?+), 9. amoxicillin (+), 10. trimethoprim (+), with negative patch test results for 2. allopurinol, 6. domperidone, and 7. cladribine. B: Positive delayed intradermal test results at 96 hours, for: 3. amikacin and 5. meropenem.

Patch test results were positive at 48 hours and 96 hours to cotrimoxazole (+), amikacin (+), piperacillin/tazobactam (++), meropenem (+), and trimethoprim (+), with additional reactions to penicillin V (?+) and amoxicillin (+). Patch tests were negative to allopurinol, domperidone, and cladribine (Figure 2A). Additional delayed-reading intradermal testing (IDT) to amikacin (25 mg/mL, 1 : 10 dilution) and meropenem (5 mg/mL, 1 : 10 dilution) were also performed at a separate timepoint to exclude spread from the strong positive piperacillin/tazobactam reaction to neighboring patch test sites (and “angry back syndrome”). These IDT to amikacin and meropenem were also positive with papular erythema at 48 hours and 96 hours (Figure 2B). Subsequent 3-day course drug provocation tests (DPT) to allopurinol (100 mg) and domperidone (10 mg) were negative. Cladribine did not undergo a DPT due to clinical risk of neutropenic sepsis recurrence and cost. She was diagnosed with delayed-type hypersensitivity (severe MPE) to piperacillin/tazobactam, cotrimoxazole/trimethoprim, amikacin, and meropenem and advised to avoid all these agents and their classes. 

## Discussion 

Here we describe a rare case of severe MPE with simultaneous MDH to piperacillin/tazobactam, cotrimoxazole/trimethoprim, amikacin, and meropenem – confirmed via delayed skin testing (positive patch test and IDT), with additional evidence of penicillin cross-reactivity. To our knowledge, this is also the first case of MDH involving these four different antibiotic agents. Notably, we deemed the meropenem reactivity to be a distinct carbapenem type IV allergy rather than beta-lactam cross-reactivity, given that negligible rates of type IV cross-reactivity have been reported [5]. 

MDH describes a syndrome of long-lasting type IV hypersensitivity to multiple different drugs following profound T-cell activation. It typically presents initially in patients with severe T-cell drug reactions (i.e., severe exanthema and DRESS) [1, 6]. Incidence of MDH in severe type IV drug eruptions is 10 – 13% [1]. Three putative subtypes of MDH have been described: simultaneous, sequential, and long-interval/distant. In simultaneous MDH, the individual becomes sensitized to different agents during the same episode of the drug reaction (i.e., at the start of therapy), whereas sequential or distant MDH occurs when subsequent different drugs are commenced days, weeks, or years later, and a second (separate) drug reaction episode occurs [1, 5, 6]. Simultaneous MDH is more likely to occur when drugs are given in fixed drug combinations, such as sulfamethoxazole-trimethoprim (cotrimoxazole/septrin), and piperacillin-tazobactam (tazocin), as seen here in our case. We propose that cotrimoxazole is likely the primary elicitor in our patient’s MDH, as it was commenced one week prior to onset, whilst the other agents preceded the MPE by 48 hours. Furthermore, in the context of the confirmed penicillin cross-reactivity (detected via patch testing), we have additionally demonstrated that the piperacillin was likely the elicitor in tazocin rather than tazobactam. Whilst full drug history for this patient was not available, antibiotics such as cotrimoxazole are commonly given in patients undergoing chemotherapy, for prophylaxis or treatment against infection, highlighting plausible earlier sensitization for development of simultaneous MDH. Other MDH risk factors include: the type of drug (i.e., sulfonamide/penicillin), high drug concentration, prolonged treatment courses, and pre-existing inflammation or immune activation such as in cancer [1]. 

MDH is not a clinical diagnosis and should be used only for well-defined type IV drug hypersensitivity reactions elicited by two or more different drugs, where their involvement has been proven on testing. Patch test and IDT are helpful diagnostic in vivo tools to confirm MDH, as are in vitro T-cell assays, and drug challenge tests (where safe) are also recommended to refine the MDH diagnosis further [1, 7]. One must also consider the possibility for “angry back syndrome” given the number of positive skin tests; however, we feel this was suitably mitigated in our case by performing separate IDTs to relevant drugs (amikacin/meropenem) showing reactivity at a different timepoint, thus confirming MDH. Further drug provocation tests to positive skin test results were not deemed ethical in the context of the severity of the widespread diffuse exanthema in our case. 

The exact mechanisms underlying MDH are not fully understood; however, the initial severe drug eruption (i.e., severe MPE/DRESS), appears to be triggered by p-i-mediated T-cell stimulation, where the drug directly binds noncovalently to a particular HLA protein itself with a strong affinity (p-i-HLA), or the drug may bind to the TCR directly (p-i-TCR) [1]. Notably, drug-reactive T-cells of MDH patients are specific to the particular drug, and they are not cross-reactive. A second important factor with MDH is massive drug-induced polyclonal T-cell stimulation and proliferation, which persists for years. Ongoing T-cell activation may act as a risk factor for other drug reactivity, and thus most likely lowers the threshold of reactivity to a second/subsequent drug [1, 8]. Interestingly, concurrent hairy cell leukemia on cladribine has also been associated with a high risk of MDH, and mechanistically this has been hypothesized to be related to abnormal lymphocyte function, lymphopenia, and subsequent decreased drug tolerance [9]. 

To conclude, we report a rare case of severe MPE in the context of hairy cell leukemia, with simultaneous MDH to cotrimoxazole/trimethoprim, amikacin, piperacillin/tazobactam, and meropenem, confirmed by patch testing and IDT. We highlight the utility of comprehensive delayed skin testing to carefully identify all drug culprits, and the importance of considering MDH as a differential diagnosis in severe type IV drug eruption cases, especially when risk factors are present. 

## Authors’ contributions 

KT and TW contributed to conception and drafting of the work. KT, CL, TB, EB, and TW contributed to acquisition of clinical data regarding the case and preparation of final manuscript. 

All authors provided final approval of work for publication. 

## Funding 

No funding was received for this work. 

## Conflict of interest 

The authors declare that they have no relevant conflict of interest.
